# Environmental impact assessment of battery boxes based on lightweight material substitution

**DOI:** 10.1038/s41598-024-53238-2

**Published:** 2024-01-31

**Authors:** Xinyu Li, Yuanhao Zhang, Yumin Liao, Guanghai Yu

**Affiliations:** 1 School of Mechanical Engineering, Hefei University of Technology, Hefei, China; 2 Intelligent Manufacturing Institute of HFUT, Hefei University of Technology, Hefei, China; 3 Anhui Province Key Laboratory of Low Carbon Recycling Technology and Equipment for Mechanical and Electrical Products, Hefei, China

**Keywords:** Mechanical engineering, Environmental impact

## Abstract

Power battery is one of the core components of electric vehicles (EVs) and a major contributor to the environmental impact of EVs, and reducing their environmental emissions can help enhance the sustainability of electric vehicles. Based on the principle of stiffness equivalence, the steel case of the power cell is replaced with lightweight materials, a life cycle model is established with the help of GaBi software, and its environmental impact is evaluated using the CML2001 method. The results can be summarized as follows: (1) Based on the four environmental impact categories of GWP, AP, ADP (f), and HTP, which are the global warming potential (GWP), acidification potential (AP), abiotic depletion potential (ADP (f)) and human toxicity potential (HTP), the environmental impact of lightweight materials is lower than that of the steel box. Among them, the aluminum alloy box has the largest reduction, and the Carbon Fiber Sheet Molding Compound (CF-SMC) box is the second. (2) In the sensitivity analysis of electric structure, an aluminum alloy box is still the most preferable choice for environmental impact. (3) In the sensitivity analysis of driving mileage, the aluminum alloy box body is also the best choice for vehicle life. (4) Quantitative assessment using substitution factors measures the decrease in greenhouse gas emissions following the substitution of steel battery box with lightweight materials. The adoption of aluminum alloy battery box can lead to a reduction of 1.55 tons of greenhouse gas emissions, with a substitution factor of 1.55 tC sb^−1^. In the case that composite materials have not been recycled commercially on a large scale, aluminum alloy is still one of the best materials for the integrated environmental impact of the whole life cycle of the battery boxes.

## Introduction

In the face of the dual challenges of global climate change and excessive energy consumption, governments worldwide have vigorously promoting electric vehicles (EVs) to achieve sustainable development, aiming to reduce greenhouse gas emissions and energy consumption^1^. Thanks to strong government support, global EV sales reached 4.3 million units in the first half of 2022, representing a year-on-year growth of 62%^2^. With the increasing adoption of EVs, the use of lithium-ion batteries, known for their high energy density and long lifespan, has become widespread and the preferred choice for EVs^3^. It is projected that global demand for lithium-ion batteries will reach 9,300 GWh by 2030^4^. However, as the number of EVs and the capacity of lithium-ion batteries continues to rapidly increase, the potential environmental impacts cannot be overlooked, and immediate action is required to mitigate their environmental pollution.

Life Cycle Assessment (LCA) is a method used to evaluate the environmental impacts of products, processes, or activities throughout their lifecycle. It plays a crucial role in quantifying environmental impacts and can be applied to assess the environmental impacts of EVs and lithium-ion batteries. Zhou et al.^5^ used this method to assess the environmental, economic, and social performance of pure EVs and gasoline vehicles, and the comprehensive results showed that pure EVs had better lifecycle sustainability assessment outcomes, indicating a more promising future. SHI et al.^6^ and ZHAO et al.^7^, based on the Chinese context, conducted scenario analyses on EVs, and the results demonstrated significant advantages of EVs over light gasoline vehicles in reducing greenhouse gas emissions. However, overall advantages do not necessarily imply advantages in all stages. The environmental benefits of EVs during the use phase are evident, while the production phase is constrained by the manufacturing of lithium-ion battery packs^8^, resulting in higher environmental impacts compared to conventional vehicles. Therefore, the overall energy and environmental performance of EVs heavily rely on whether the advantages during the use phase outweigh the additional impacts caused by the production of lithium-ion battery packs. Reducing the environmental impacts of lithium-ion battery pack manufacturing is crucial for enhancing the overall environmental benefits of EVs.

Among the entire lithium-ion battery pack, the battery enclosure, which protects the vehicle body system and ensures electrical safety, exhibits the highest environmental emissions throughout the production phase, accounting for up to 63% ^9^. The proportion of environmental emissions from battery boxes varies among different types of lithium batteries, influenced primarily by the extraction of various cathode materials and the assembly of battery packs using different technological processes. Both factors result in fluctuations in environmental emissions due to resource and technological limitations, which are difficult to mitigate in the short term. Excluding these factors, the proportion of emissions from battery boxes is at least 30%^3^. Therefore, reducing the environmental impacts of battery boxes can effectively enhance the environmental benefits of lithium-ion battery packs.

Lightweighting, as one of the measures for energy saving and emission reduction in automobiles, is widely applied to automotive components such as seats^10^, engine hoods^11^, and fenders^12^. Research on lightweighting in combination with battery boxes has also been conducted. Kaleg et al.^13^ selected the 5052–0 series aluminum alloy as the material for the battery enclosure and, through finite element analysis, determined that a material thickness of 2 mm and a mass of 6.51 kg were the optimal design for the battery pack. Lan et al.^14^ achieved structural lightweighting of the power battery enclosure through the use of multiple materials and a reasonable structural design, resulting in a weight reduction rate of 47.3%. Studies have shown that for every 1 kg reduction in the curb weight of an electric vehicle, energy consumption decreases by 0.0051 kWh^15^. Lightweight materials can significantly reduce energy consumption and environmental emissions during the use phase of electric vehicles. However, the advantages during the use phase may not necessarily offset the disadvantages during the manufacturing phase. A detailed analysis of the specific situation is necessary. Currently, there is no specific LCA study on lightweight battery boxes. In studies on the full lifecycle of electric vehicles or power batteries, when the battery enclosure is considered, typically only the main materials are included in the life cycle assessment, without considering the use of other auxiliary materials and the energy consumption of related processes, especially the coating process of the battery enclosure, which contributes a considerable proportion to the overall environmental emissions^16^.

The battery enclosure, as a structural component of a power battery, has significant potential for lightweight design and energy-saving and emission reduction. This paper focuses on the steel battery enclosure of a specific automobile and explores the substitution of aluminum alloy and CF-SMC (Carbon Fiber Sheet Molding Compound) composite materials. A "cradle-to-cradle" life cycle model is constructed based on production data of these materials to assess various environmental impact potentials. By comparing the environmental impacts of the steel battery enclosure with those of lightweight materials such as aluminum alloy and CF-SMC composite material battery boxes, this study provides an environmental decision-making basis for selecting raw materials for battery boxes and offers partial references for the overall life cycle assessment of lithium-ion battery packs.

## Method

According to the ISO 14,044 standard, the Life Cycle Assessment (LCA) framework consists of four main parts: goal and scope definition, life cycle inventory, impact assessment, and result interpretation^17^.

### Goal and scope definition

GaBi software, a commonly used tool for LCA, incorporates various established life cycle impact assessment methods, which facilitate the calculation and evaluation of multiple environmental indicators. Among these, the CML2001 method is highly regarded and extensively applied due to its comprehensive algorithmic structure, ensuring the comparability and rigor of research results, thus meeting the standard requirements of life cycle assessment quite effectively. In this study, a cradle-to-cradle life cycle model of battery box was constructed using GaBi software, and the environmental benefits of different material battery box were analyzed and compared using the CML2001 method.

#### Evaluation object and functional unit

This study focuses on comparing three battery boxes: a base case steel battery enclosure (1400 mm × 1200 mm × 200 mm), and two alternative lightweight materials: aluminum alloy and CF-SMC. The functional unit is defined as the entire battery enclosure, ensuring a quantitative assessment and comparability of carbon emissions throughout the entire life cycle of different material battery boxes.

#### Data sources

Collecting production data for a product is often challenging in LCA studies, as reliable data from the industry is often scarce, especially in the lithium battery industry with technological barriers. In this study, grey literature, specifically environmental impact assessment reports from representative manufacturers in China, was chosen as the source of production data. These reports provide detailed information on planned facilities, including annual production capacity, raw material and energy requirements, and estimates of on-site pollutant emissions. A top-down approach was used to obtain accurate and reliable production data.

For basic materials where upstream production data is unavailable (such as electricity, welding wire, compressed air, etc.), and considering the lack of a publicly available and comprehensive life cycle database in China, data primarily obtained from the built-in database of the GaBi software.

Moreover, the system boundary does not include the resources, energy consumption, and labor consumption associated with the manufacturing, introduction, maintenance, and depreciation of production machinery.

The mass of battery boxes made from different materials was determined based on the principle of equivalence in terms of specifications and rigidity^18^, as detailed in Table 1.

**Table 1:** Battery pack box quality and data source.

Materials	Mass/kg	Foreground data			Background data	
		Raw materials	Energy consumption	Outputs	Production of materials and energy sources	Indirect emissions
Q235	97.22	Anhui^19^			GaBi database^22^ and related literature^19–23^	
AL6061	49.61	Guangzhou^20^				
CF-SMC	34.56	HEXCEL^21^				

#### System boundary

**Figure 1:**
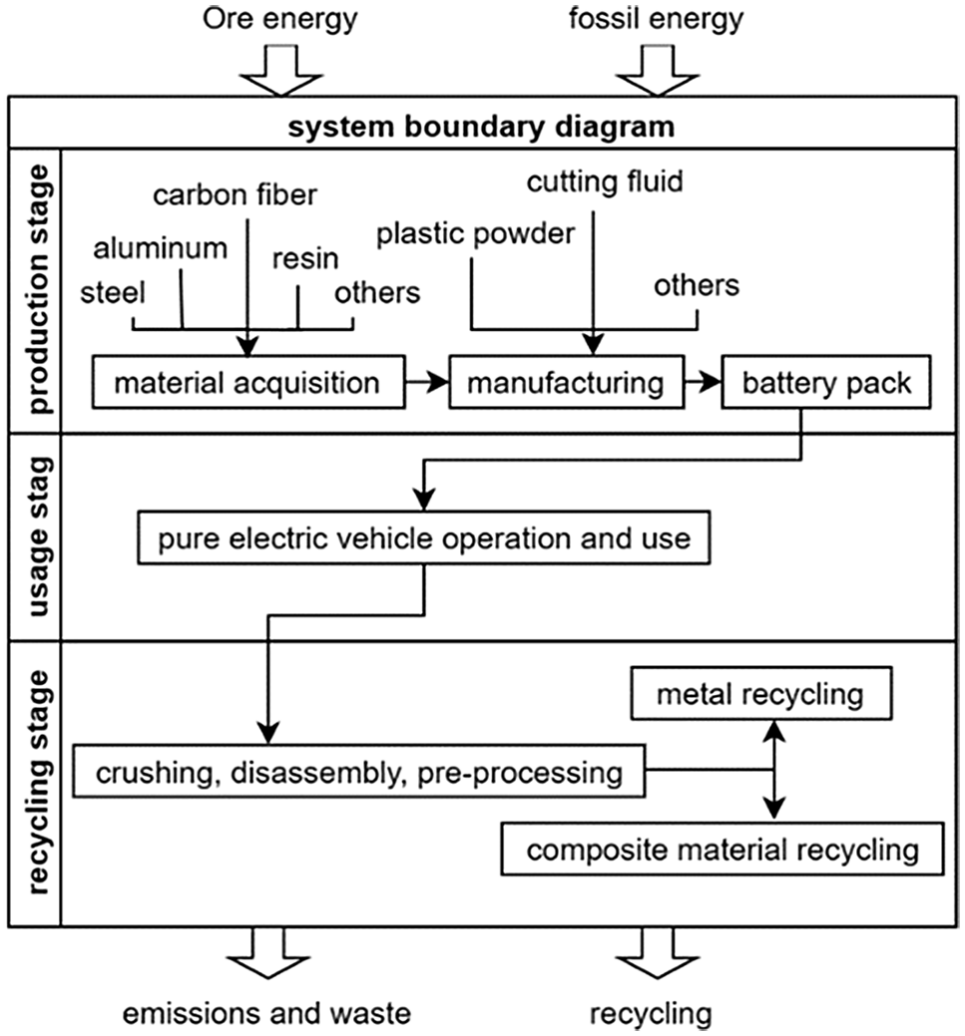
System boundary.

The system boundary, as depicted in Fig. 1, encompasses the entire life cycle system, incorporating the production stage, use stage, and recycling stage. However, due to the difficulty in obtaining data regarding product transportation and storage, these aspects are not included in the scope of this study.

### Life cycle inventory

A life cycle inventory analysis involves the compilation and quantification of input and output flows for a given product system throughout its entire lifecycle or individual processes. These flows consist of inputs such as water, energy, and raw materials, as well as outputs of products and environmental emissions. Environmental emissions can be categorized into direct emissions and indirect emissions. Direct emissions refer to the emissions released directly into the environment during a specific stage, which can be obtained through measurement data. On the other hand, indirect emissions refer to the environmental emissions associated with the production of materials/energy inputs, requiring tracing back to their sources. Supplementary data for these emissions can be obtained from the GaBi software database,as detailed in Table 1.

#### Production stage inventory

According to a study by Linda^24^, significant variations in environmental impacts caused by different stages of battery pack production are primarily due to different assumptions made regarding the energy associated with battery manufacturing and pack assembly. To mitigate the influence of such variations, the electrical energy consumed in battery pack production is assumed to be sourced from the China Mix Electricity dataset in the GaBi database.

Moreover, to enhance comparability among the three factors, the material and energy values should approach an ideal state, excluding any human or mechanical interference.

Regarding materials: The qualified rate of battery pack production is assumed to be 100%, meaning the inventory quantity of materials is calculated as the total amount minus the average amount of defective products. The waste packaging of materials is assumed to be recycled by the manufacturer and is not considered in the calculation. The quantity of materials represents the actual amount used. For example, for cut-off scraps in battery pack production, it is assumed that they can be recycled. However, for consumables such as welding wire, the quantity is calculated by subtracting the amount of welding slag generated. The same principle applies to water, where the total usage amount is subtracted by the amount of recycled water.

**Figure 2:**
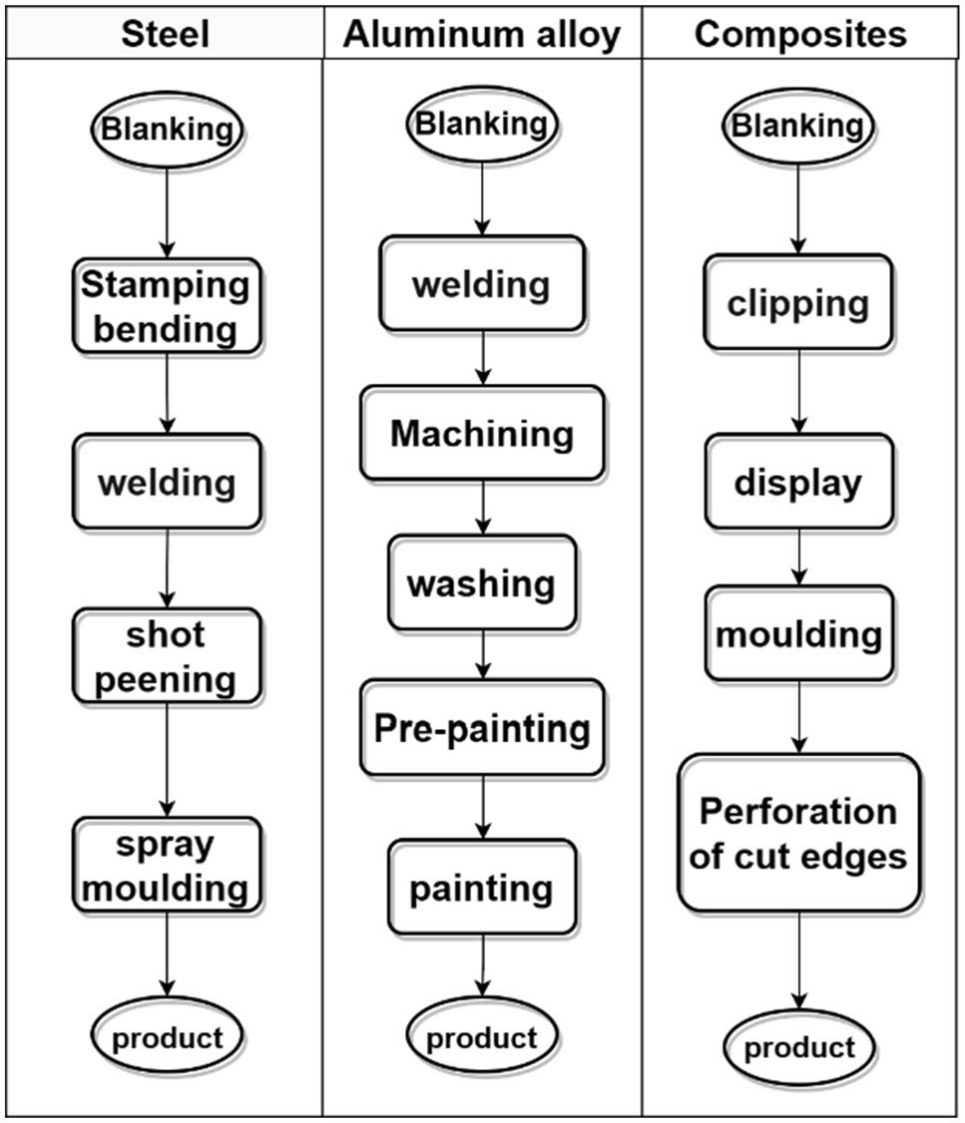
The main energy consumption process flow diagram.

Regarding energy: The energy consumption, mainly electrical energy, associated with the battery pack production stage in the environmental impact assessment report lacks detailed information, including household electricity consumption unrelated to the production process. Therefore, to obtain a more realistic inventory of the production stage, the primary energy-consuming processes in Fig. 2 and the unit process life cycle inventory (UPLCI) are combined to quantify the energy consumption in the manufacturing of battery packs.

As depicted in Fig. 2, the production stage of the steel battery pack comprises four primary production units: stamping and bending, welding, shot blasting, and powder coating. The UPLCI for stamping and bending, as well as powder coating, can be obtained from the GaBi database, with a total energy loss of 6.67 kWh. Shot blasting involves the high-speed projection of pellets onto the surface of the welded workpiece, resulting in a smooth surface through the collision between the pellets and the workpiece. This process aims to remove rust and enhance the adhesion rate of solid powder. The energy consumption for shot blasting is determined by referring to the power and time of the shot blasting machine provided in the environmental impact assessment report^19^, totaling 0.53 kWh.

For the welding of steel battery packs, carbon dioxide gas shielded welding or resistance spot welding is commonly employed. Here, we take the former as an example and determine the electricity consumption based on the actual consumption of welding materials^25^, which amounts to 0.0996 kWh. Please refer to Table 2 for further details.

**Table 2:** List of production stages of steel battery box.

Categories	Name	Unit	Amount
Materials	Q235B Q345E	kg kg	5.07 × 10^1^ 3.54 × 10^1^
	welding wire	kg	4.98 × 10^–2^
	Carbon dioxide	kg	2.78 × 10^–3^
	Epoxy resin—thermosetting	kg	2.23 × 10^–1^
	Oxygen	kg	5.55 × 10^–2^
	Lubricating oil	kg	6.94 × 10^–3^
	Shot peening media Steel fittings	kg kg	2.72 × 10^–3^ 1.09 × 10^1^
Energy	Electricity	kwh	7.25 × 10^0^
Process emissions	PM10	kg	9.20 × 10^–3^
	Vocs	kg	3.17 × 10^–4^

Presently, domestically manufactured aluminum alloy battery packs are primarily produced using a combination of friction stir welding and a small amount of arc welding process^26^. The primary material utilized is 6061 aluminum profiles, which exhibit low comprehensive application costs and meet the required performance standards. Given that most of the processes are concentrated on the lower part of the battery pack^20^, the upper part only necessitates simple machining, thus the energy consumption is encompassed within the machining process of the lower part. As depicted in Fig. 2, the energy consumption processes for the battery pack primarily pertain to the production of the lower part. The UPLCI for the last four production units is derived from the GaBi database, resulting in a total energy consumption of 15.852 kWh.

The welding process for the production unit unfolds as follows: initially, the bottom plate is joined using friction stir welding, while the beams are welded together using argon arc welding to form a complete frame. Ultimately, the bottom plate and frame are double-sided welded using friction stir welding, with an electricity consumption of 41.2 kWh^27^. The inventory of the production stage is displayed in Table 3.

**Table 3:** List of production stages of aluminum profile battery box.

Categories	Name	Unit	Amount
Materials	Aluminum 6061	kg	4.68 × 10^1^
	Cleaning agents	kg	2.14 × 10^–2^
	PVC coating	kg	2.10 × 10^0^
	Welding wire	kg	2.06 × 10^–1^
	Cutting oil	kg	9.02 × 10^–3^
	Argon gas	kg	4.40 × 10^–1^
	Steel fasteners	kg	4.87 × 10^–1^
	Water	kg	4.54 × 10^1^
Energy	Electricity	kwh	5.71 × 10^1^
Process emissions	COD	kg	7.68 × 10^–5^
	NH	kg	9.60 × 10^–6^
	PM10	kg	1.15 × 10^–2^
	Vocs	kg	1.13 × 10^–3^

Carbon Fiber Sheet Molding Compound (CF-SMC) is a material that comprises of dispersed carbon fiber bundles in a matrix material. In comparison to traditional Sheet Molding Compound (SMC), CF-SMC possesses greater strength and enables effective reduction in component thickness. Moreover, the compression molding process of CF-SMC can significantly reduce molding time, labor costs, and facilitate low-cost, high-performance, and efficient mass production of carbon fiber products.

The initial production unit of the CF-SMC battery pack is known as "preparation," encompassing the preforming of carbon fiber and CF-SMC. The production of 1 kg of carbon fiber necessitates the consumption of 2.08 kg of polypropylene and 460.7 MJ of primary energy^35^. The CF-SMC product offered by Hex company^21^ is composed of 62% carbon fiber and 38% unidirectional prepreg (resin). The compression molding, demolding, and trimming processes can be referenced from the traditional SMC compression molding process^28^. The total energy consumption for the four production units amounts to 693.2 kWh. The production inventory is provided in Table 4. The emissions resulting from primary energy sources such as natural gas and petroleum are calculated using GaBi software. Within the inventory, the process emissions primarily originate from the compression molding and trimming processes.

**Table 4:** List of production stages of carbon fiber battery box.

Categories	Name	unit	amount
Materials	Polyacrylonitrile	kg	4.46 × 10^1^
	Natural gas	kg	1.17 × 10^2^
	Coal	kg	1.22 × 10^1^
	Petroleum	kg	6.04 × 10^1^
	UD prepreg	kg	1.34 × 10^1^
	Hydraulic oil	kg	5.41 × 10^–2^
	Lubricants	kg	3.61 × 10^–3^
Energy	Electricity	kwh	6.93 × 10^2^
Process emissions	Vocs PM10	kg kg	7.75 × 10^–3^ 2.52 × 10^–3^

#### Use phase inventory

The utilization of the battery pack is dependent on the power battery, and the use of the power battery is reliant on new energy vehicles. Hence, to enhance the use phase inventory, it is necessary to establish specific scenarios for the power battery and new energy vehicles when calculating the utilization of the battery pack. Simultaneously, the indirect emissions associated with the use phase are influenced by the battery conversion loss, energy required for battery weight transportation, and the carbon intensity of electricity^24^. The electricity assumption remains consistent with the production phase, while transportation consumption is not considered in this study. The battery conversion loss is assumed to have a fixed efficiency, serving two primary purposes. First, it simplifies the research scenario, allowing a more focused analysis of the energy consumption of the battery box without being affected by factors such as reduction of conversion efficiency and capacity degradation due to battery aging. Second, it eliminates the influence of human or mechanical intervention, enhancing the reliability and comparability of the evaluation results by ensuring that the data approaches an ideal state. The specific assumptions are outlined as follows:

The total capacity of the power battery is 40 kWh, with a power consumption rate of 9.7 kWh per hundred kilometers. The charging efficiency is 92%, and the discharging efficiency is 90%. The number of charging and discharging cycles is set at 5000. According to China's "Mandatory Scrapping Standards for Motor Vehicles," the total mileage of new energy vehicles is established as the theoretical scrapping limit, which amounts to 600,000 km, based on the energy loss between the power battery and the drive system. The mass of the vehicle, excluding the battery pack, is 1500 kg. The energy consumption of the battery pack during use is allocated to the power battery usage phase utilizing the principle of mass allocation^29^. The calculation formula is presented as follows:

$${{\text{E}}}_{{\text{ev}}}=\frac{Q\cdot L\cdot {m}_{1}}{\mu \cdot \gamma \cdot 100\cdot ({M}_{2}+{m}_{1})}$$

In the equation, ${{\text{E}}}_{{\text{ev}}}$ represents the energy consumption during the use phase of the new energy vehicle battery pack, $Q$ represents the power consumption per hundred kilometers (kWh), $L$ represents the driving distance of the new energy vehicle (km), $\mu$ represents the charging efficiency, $\gamma$ represents the discharging efficiency, ${m}_{1}$ represents the mass of the battery pack (kg), and ${M}_{2}$ represents the mass of the entire new energy vehicle excluding the battery pack (kg). The calculation results are shown in Table 5.

**Table 5:** List of battery pack boxes in use stage.

Categories	Unit	Energy consumption
Steel	kwh	4278.42
Aluminum	kwh	2250.27
CF-SMC	kwh	1583.01

#### Second use stage

When the capacity of a power battery decreases to below 80%, it is considered retired in the field of new energy vehicles. However, apart from the decrease in capacity, the battery still maintains its other functionalities. Therefore, retired batteries can be collected and reused in other fields, primarily in the energy storage sector. In order to repurpose the retired automotive battery pack into an energy storage system, the original battery casing needs to be dismantled and replaced with a new casing suitable for the energy storage system. The dismantled battery casing can be reused in the same vehicle model, thus enabling a second use stage. The energy consumption during this stage is determined by the mileage driven and is considered an extension of the usage stage. However, if the battery casing remains intact, it can be reused multiple times. Due to the lack of relevant data, this stage is not included in the life cycle assessment but is discussed based on the increase in mileage as a reference for exploring the related environmental impacts. For more details, please refer to Fig. 6.

#### Recycling stage inventory

Due to the incomplete recycling process for battery packs at present, the recycling of primary materials is adopted here to simplify and substitute for the recycling process. The recycling rates for traditional metal steel and aluminum alloys are 90% and 92%, respectively^30^. "Cradle to Cradle" represents a closed-loop life cycle where materials recycled at the end of one cycle re-emerge as raw materials for the next. This approach reduces the demand for raw materials, resulting in a positive environmental impact^31^. The recycled metals will be used in the production phase, which will have beneficial environmental effects.

CF-SMC belongs to thermosetting composite materials. Due to their excellent strength-to-weight ratio, thermal characteristics, and thermal stability, they are preferred materials in many automotive applications. However, there is an objective difficulty in recycling them compared to traditional metal materials, which can be easily recycled through simple remelting. CF-SMC materials require different recycling methods for processing.

Currently, there are four main types of recycling technologies for thermosetting composite materials: energy recovery, physical recycling, chemical recycling, and thermal decomposition. Each method has its own advantages and disadvantages. Thermal decomposition involves heating to volatilize the resin and remove it, with by-products typically being gases and liquids from solid and inorganic materials in the composite. It is suitable for bulk composite waste processing and recycling, with minimal loss of mechanical properties in the recycled material. It is one of the few waste recycling methods that has achieved industrialization. Furthermore, the liquids and gases generated during the thermal decomposition of composite materials can be used as fuel in the process, reducing thermal consumption^32^.

During the thermal decomposition of CF-SMC, the gasified resin is burned, and the energy generated by the combustion can meet the thermal consumption, with the remaining energy having a positive impact on the environment elsewhere. For detailed pyrolysis methods, the patent for Carbon Fiber Recycling Company should be consulted^33^. Inventory data can be found in the supplementary materials. After thermal decomposition, regenerated carbon fiber with a shorter length, minimal loss of mechanical properties, and a smooth and clean surface is obtained, with a net greenhouse gas emission of 0.573 kg-CO2eq/kg^34^. Due to the loss of mechanical properties, the regenerated carbon fiber will not be used in the original production phase but in the manufacturing of other carbon fiber components, with the resulting environmental impact not accounted for in the original process.

## Results

**Figure 3:**
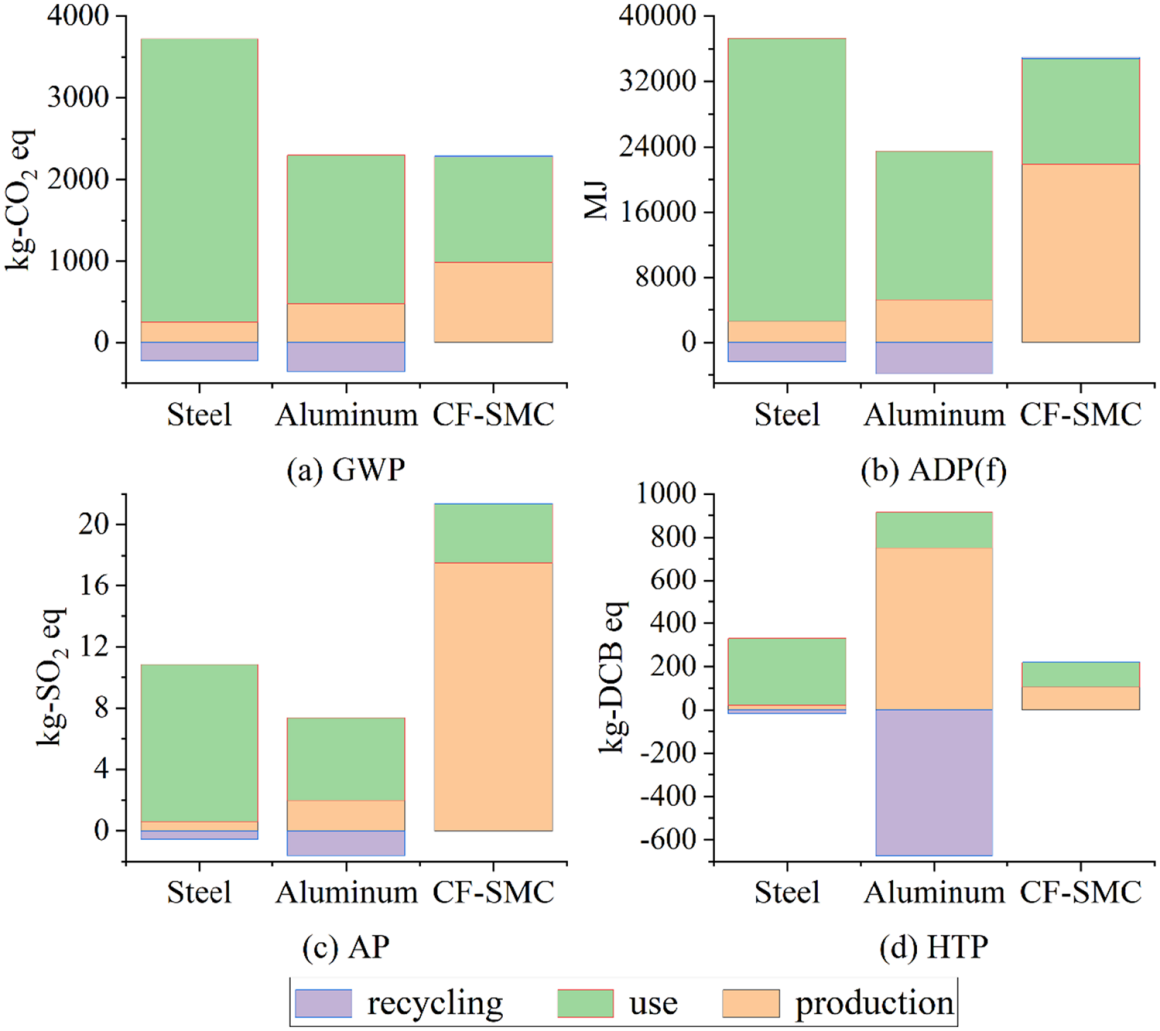
Life cycle assessment results.

The results of the life cycle assessment part shown in Fig. 3 were generated using the CML2001 method from the life cycle inventory of the battery pack. Four representative environmental impact categories were selected: global warming potential (GWP), abiotic depletion potential of fossil resources (ADP(f)), acidification potential (AP), and human toxicity potential (HTP). To facilitate the comparison of different battery packs, the subsequent text will use material codes to refer to the packs.

### Life cycle impacts

Figure 3(a) first considers the global warming potential (GWP) in kilograms of carbon dioxide equivalent. The steel battery casing has the highest environmental emissions among the three materials, with the usage stage accounting for over 90% of the total emissions. It can be observed that the GWP during the usage stage decreases with a decrease in casing weight, reflecting the advantages of lightweighting. The GWP during the usage stage for aluminum alloy and CF-SMC decreases by 47.4% and 63.0%, respectively. In the production stage, the emissions of lightweight materials increase compared to steel, with aluminum alloy increasing by 90% and CF-SMC increasing nearly threefold. In the recycling stage, metal materials have negative values while composite materials have positive values. The recycling process for metal materials is more mature compared to composite materials, and the recycling and reuse approach can reduce overall emissions. When considering the carbon dioxide equivalent over the entire life cycle, aluminum alloy and CF-SMC reduce emissions by 44.4% and 34.6%, respectively. Although CF-SMC saves the most weight, it achieves less emission reduction compared to aluminum alloy. Overall, aluminum alloy has the lowest GWP and the least global warming impact.

Figure 3(b) considers the non-biological depletion of fossil fuel potential in megajoules (MJ), quantifying the impact on resource extraction and non-biological energy sources, which represents the depletion of natural fossil fuel resources. In the usage stage, the values for these materials in this impact category are proportionate to those in Fig. 3(a), as the energy consumption during the usage stage is consistent in China. In the production stage, the increment for aluminum alloy is similar to the increment in GWP, while CF-SMC sees a significant increase of 764%. Despite the increase in environmental burden during the production stage in the life cycle, all lightweight materials still achieve a reduction compared to steel in this impact category. The impact of CF-SMC is limited compared to steel, with a difference of only 43.74 MJ, while aluminum alloy reduces by 43.9%, approximately 15,000 MJ.

The acidification potential of the battery casing is shown in Fig. 3(c), measured in kilograms of sulfur dioxide (SO2) equivalent. Acidification potential is an indicator of potential soil and water acidification due to the release of gases such as nitrogen oxides and sulfur oxides. Similar to other impact categories, the use of lighter materials reduces the impact during the vehicle usage stage. In the production stage, CF-SMC has the highest impact, followed by aluminum alloy and then steel. In terms of the total impact in this category, aluminum alloy still has the lowest impact (5.754 kg-SO2 eq), but the highest impact is not steel, but CF-SMC (21.378 kg-SO2 eq). The low impact of aluminum alloy is due to the offset in the recycling stage, where the recycling and reuse of aluminum alloy greatly reduce the acidification potential. The production stage of CF-SMC, which uses coal and natural gas, contributes to the highest acidification potential.

Human toxicity potential refers to the impact of toxic substances emitted into the environment on humans, and it is divided into non-carcinogenic and carcinogenic toxic substances. The results of the impact are shown in Fig. 3(d). Aluminum alloy has the lowest overall impact, followed by CF-SMC, and steel has the highest impact. Among them, the production stage of aluminum alloy has the highest impact, which is 35.7 times that of steel, but the recycling stage effectively reduces this impact.

In conclusion, aluminum alloy battery casings outperform steel and CF-SMC in terms of four environmental impact categories. Steel has the highest impacts in terms of GWP, HTP, and ADP(f), while it is second to CF-SMC in terms of AP.

### Production stage emission analysis

**Figure 4:**
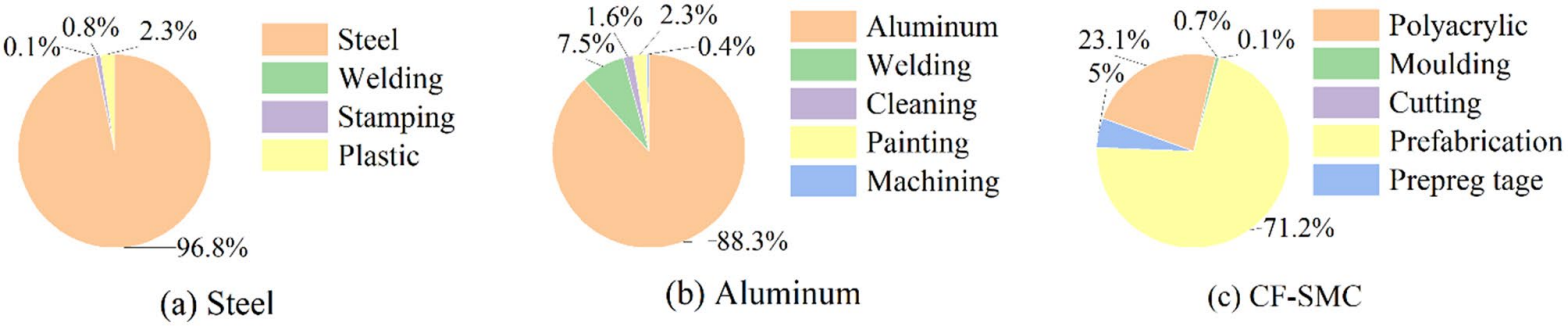
Main emissions in the production stage of steel, aluminum alloy and CF-SMC.

An analysis of emissions during the production stage of the product life cycle was conducted, focusing on the global warming potential (GWP) as an example. It was found that replacing the battery casing with lightweight materials leads to higher environmental emissions during the production stage. To effectively improve environmental benefits, it is crucial to understand the factors contributing to these increases. Contribution analysis was conducted for the initial production life cycle stage to identify the materials and processes with the highest contributions to CO2 equivalent emissions and changes. Figure 4(a)-(c) illustrates the major emissions during the production stage for steel, aluminum alloy, and CF-SMC, with total emissions of 247.46, 471.92, and 989.14 kg-CO2 eq, respectively.

In this scenario, aluminum alloy and CF-SMC casings perform poorly in terms of lifecycle CO2 equivalent emissions. The process contributions of aluminum alloy casing components are shown in Fig. 4(b). The production of aluminum alloy dominates, accounting for 88.3% of the emissions, while the remaining processes contribute 11.7%. For CF-SMC, the production of polypropylene resin contributes approximately 23.1% of the emissions, with the largest contribution coming from the preforming operation during the production process, accounting for 71.2%. If carbon fiber is included as one of the component materials, it will encompass polypropylene resin and preforming, resulting in a contribution of 94.3%. The main contribution of steel casing is from the steel material itself, accounting for 96.8% of the emissions. The primary emissions of all casings occur during the material stage, indicating that a rational and efficient recycling method is a powerful tool for reducing these emissions.

### Sensitivity analysis

Sensitivity analysis helps identify key factors that influence the evaluation results and assess the extent to which these factors impact the evaluation results when they change. The main sensitivity factors chosen here are the power structure and driving mileage.

#### Power structure

Different power structures result in different environmental impacts. In 2020, China's power structure was dominated by thermal power, which consists of coal-fired power, oil-fired power, and gas-fired power, with coal-fired power being the primary source. In the process of moving towards "peak carbon and carbon neutrality," China's power structure will inevitably become greener and low-carbon. According to a report from the China Electricity Council, the projected power structure for 2050 is shown in Table 6.

**Table 6:** Electrical structure.

Categories	2020 (%)	2050 (%)
Thermal power	67.9	15.0
Hydropower	17.0	9.0
Wind power	6.0	38.5
Photovoltaic	3.5	21.5
Others	5.6	16.0

**Figure 5:**
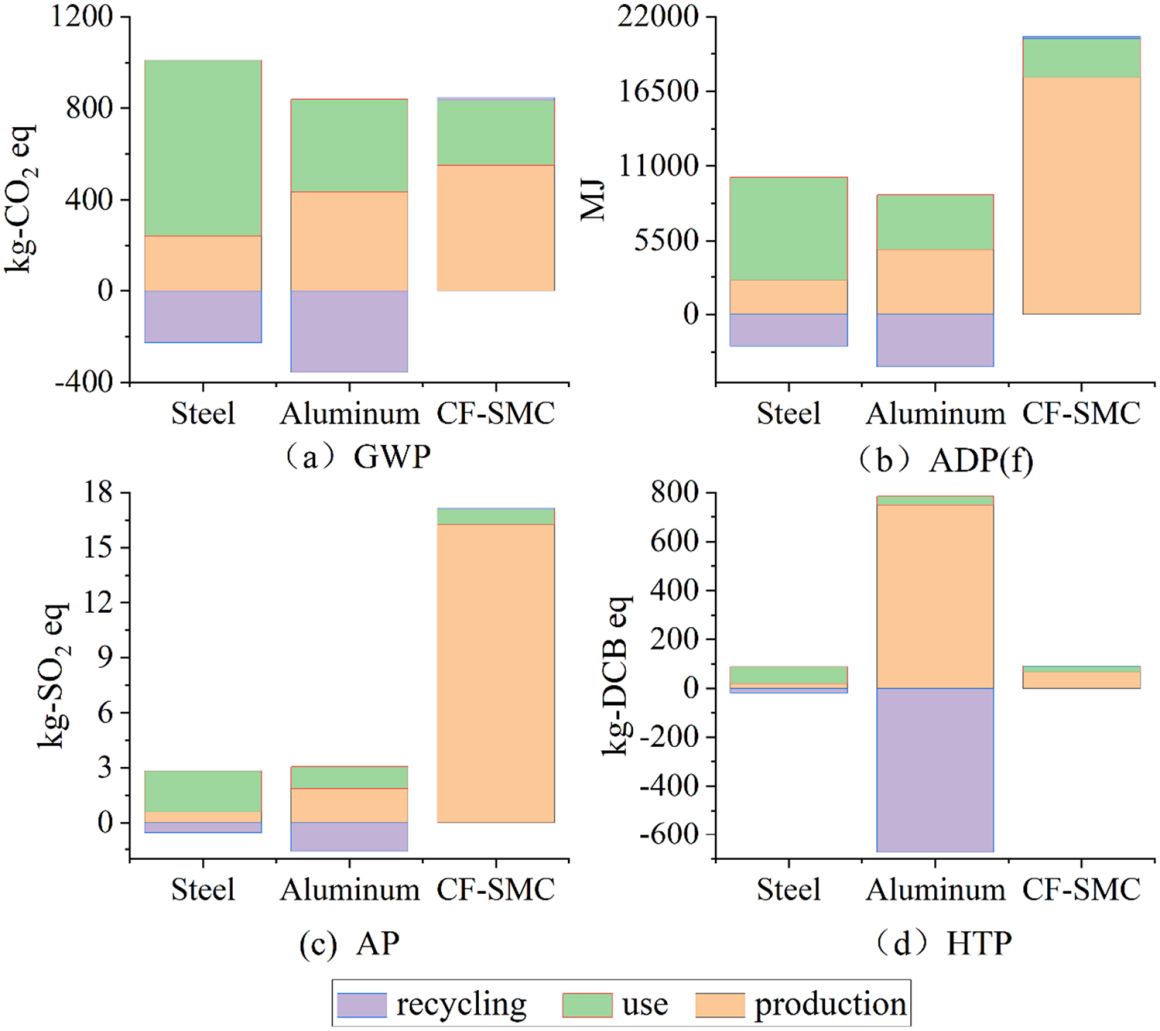
Environmental impact of electrical structure in 2050.

The results can be obtained from Fig. 5. It is evident that the environmental impact is smaller under the power structure scenario of 2050. During the production stage, the more electricity is used, the greater the impact. Steel has a smaller reduction, while aluminum alloy and CF-SMC have larger reductions. Apart from a decrease of 0.43% for aluminum alloy excluding HTP, the reductions for the rest are around 7%. CF-SMC shows reductions of 44.34%, 20.02%, 7.27%, and 37.37% in the four impact categories, respectively. The use stage still experiences a reduction in comparison. The impact during the recycling stage is relatively minor. However, when considering the entire lifecycle, there is a significant decrease in the environmental impact of all casings, predominantly reflected in the reduction during the use stage. If we only consider the impact caused by future changes in the power structure, aluminum alloy casings will still be the environmentally optimal choice in 2050.

#### Driving distance

**Figure 6:**
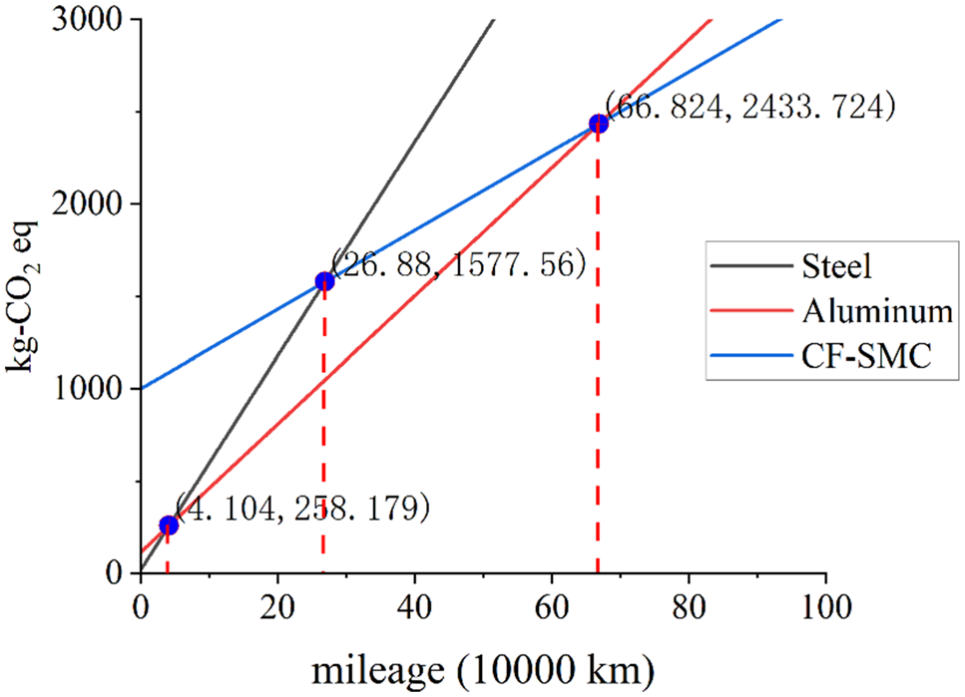
Environmental impact analysis of mileage.

Unlike the multiple impacts of power structure, the driving distance only affects the energy consumption during the use stage. Due to the uniformity of energy sources, the environmental impact values in the four impact categories during the use stage are distributed proportionally. Therefore, it is only necessary to analyze the impact of the driving distance from a single impact category. As shown in Fig. 6.

Before reaching a driving distance of 41,040 km, steel casings have the most favorable environmental impact, while CF-SMC casings have the highest impact. It is not until a driving distance of 668,240 km that aluminum alloy casings maintain the lowest impact during this period equivalent to CF-SMC casings. Afterward, regardless of the driving distance, CF-SMC casings will always have the lowest GWP among the four. Whether it is the lifespan of new energy vehicles or the current actual lifespan (about 200,000 km), aluminum alloy casings are undoubtedly the environmentally optimal choice. Moreover, within the scope of the actual lifespan, steel casings are superior to CF-SMC casings. If considering the secondary or multiple uses of battery casings, carbon fiber casings will become the environmentally optimal choice when the cumulative driving distance reaches 668,240 km. In addition, under the influence of the power structure in 2050, CF-SMC casings are expected to have an equivalent environmental impact to aluminum alloy casings at a driving distance of 380,000 km.

In summary, if battery casings are not reused, aluminum alloy materials are more suitable for the vehicle lifespan, as the suitable driving distance for steel is too low and CF-SMC exceeds the currently defined vehicle lifespan in the market.

## Discussion

In the above study, a life cycle assessment of battery box made from three different materials was conducted to analyze their environmental impacts in practical applications.

### Impact of material quality

The results indicate that lightweight materials, such as aluminum alloy and CF-SMC, generally have lower environmental impacts compared to steel box. However, the magnitude of the impact reduction or increase does not solely depend on the material quality. On one hand, aluminum alloy, which is of lower quality, has the lowest environmental impacts in four categories. On the other hand, carbon fiber, which has the lowest quality but advantages in the usage stage due to its low weight, has the smallest impact in all four categories. However, its emissions during the production stage cannot be ignored and rank high in the four categories, completely offsetting the advantages in the usage stage. Therefore, weight reduction is not a reliable single indicator for improving environmental performance. The environmental performance of lightweight components is strongly influenced by the materials used. The use of lighter, high-performance materials may increase the environmental burden in the production stage, limiting the effectiveness of weight reduction to varying degrees. For example, the production stage of CF-SMC box has a significant impact on the environment, exceeding the emission reduction achieved through weight reduction and resulting in a net increase in environmental impacts. Aluminum alloy box also exhibit this phenomenon, but due to higher recycling rates, their lifecycle emissions are lower.

### Uncertainties and limitations

The quality of results in such studies largely depends on the availability of input data, which must be ensured to be up-to-date and representative of the assessed technologies; otherwise, the results may be highly uncertain. Firstly, for any specific LCA study, fully localized background databases are always preferred. In this study, relevant data from the environmental impact assessment report of domestically produced battery box in China were used for the production stage. However, the upstream traceability of some materials inevitably relies on industrial data from abroad, which may not fully align with actual data in China, introducing some bias to the study results. Moreover, data for the recycling stage were derived from the GaBi database, and some data may be outdated. There is also significant uncertainty in the use of materials (such as solvents) and energy in the recycling stage. Additionally, the usage stage did not consider various factors under complex backgrounds, such as the relevant impacts generated by infrastructure (e. g., charging stations, roads). Thus, there is uncertainty propagated in each stage's background dataset, but these data contribute relatively little to the final results. These basic settings in the modeling certainly introduce some uncertainties, which are difficult to avoid. In conclusion, the uncertainties in this study are within an acceptable range, and it is hoped that more reliable data can be obtained in the future to achieve more accurate results.

The study's findings are constrained by several assumptions made in the existing inventory and analysis process. Initially, the calculations utilized a constant battery conversion efficiency, neglecting the decline in efficiency as mileage increases in real-world conditions. This oversight may introduce bias into the assessment of the environmental impacts during the time-of-use phase. Subsequent research will incorporate varied conversion efficiencies to ensure a more precise analysis. Additionally, the system boundaries fail to encompass the complete life cycle phase, omitting aspects such as transportation and distribution. Furthermore, although the potential reuse of battery boxes was briefly addressed, it lacked a comprehensive model, consequently restricting the evaluation of their long-term environmental impacts. Notwithstanding these limitations, the study delivers a valuable comparative assessment of different battery box materials and provides insights into potential improvement opportunities that can be instrumental in decision-making processes. A more comprehensive analysis is imperative to achieve a thorough understanding of the environmental performance of battery boxes throughout their life cycle.

### Substitution factor

This study introduces an substitution factor for assessing the decrease in greenhouse gas emissions resulting from the substitution of steel battery boxes with lighter materials^36^. The unit, represented as t CO_2_ eq/steel box and abbreviated as tC sb^−1^, indicates the amount of greenhouse gas emissions avoided for each functional unit of a steel battery box after material substitution. According to the results of the life cycle analysis, the product substitution factor for aluminum alloy battery box is 1.55 tC sb^−1^, meaning that the production of each aluminum alloy battery box can reduce approximately 1.55 t CO_2_ eq emissions. Similarly, the product substitution factor for CF-SMC battery box is 1.21 tC sb^−1^, indicating that the production of each CF-SMC battery box can reduce approximately 1.21 t CO_2_ eq emissions.

These substitution factors offer crucial insights for decision-making, enabling the quantification of the environmental advantages associated with the use of lightweight material battery boxes and lending support to the transition to low-carbon development.

### Prospects for composite materials

The main reasons for the environmental burden of composite materials are the production and recycling of raw materials. Due to the higher thermal demand in the production stage's curing and forming processes and longer cycle times, the manufacturing or conversion stage is also more energy-intensive compared to steel, resulting in greater environmental impacts and costs. Substituting steel with any lighter material will make the manufacturing process more dominant in the product's life cycle. Therefore, developing new processes for material production and recycling is still the most effective way to reduce the material's energy consumption and emission burden^37^.

Currently, the focus of composite material recycling is on the separation and reuse of material components^38^. Recycled materials typically experience reduced mechanical and thermal properties following recycling treatment. However, the addition of additives has the potential to enhance the mechanical properties and thermal stability of recycled materials. Furthermore, thermoplastic materials offer improved recyclability compared to commonly used thermosets, as they can be more easily reprocessed and remolded. Future developments in composite recycling processes will emphasize the use of new environmentally friendly additives to enhance the properties of recycled materials, as well as the exploration and adoption of new thermoplastic materials to replace traditional thermoset materials.

If the recycling processes for composite materials have been improved to enable product reuse and achieve high recycling rates, the comprehensive application of composite materials in automobiles will be not far off from an environmental benefit perspective.

## Conclusion

In this study, a life cycle assessment of three battery boxes was conducted, and the following results were obtained:

1. Steel box have the highest environmental impacts in terms of GWP, HTP, and ADP(f), and are second only to CF-SMC in terms of AP. Aluminum alloy box are more environmentally friendly in the four categories. Reducing the weight of automotive components does not necessarily improve environmental performance. The environmental burden during the production stage of lightweight materials offsets the environmental benefits in the usage stage.
2. Increasing the proportion of green power in the power structure can reduce carbon emissions. It is predicted that by 2050, the environmental emissions of the three battery boxes will all decrease. CF-SMC box have the largest reduction in the four impact categories, while steel box have the smallest reduction. However, aluminum alloy box still have the smallest environmental impact.
3. Between 41,040 km and 668,240 km, aluminum alloy box are the most suitable choice for the lifespan of automobiles, and the environmental benefits of metal materials are higher than those of composite materials before reaching a mileage of 268,800 km. Considering the multiple reuse of battery boxes, CF-SMC box will be the optimal choice for reducing environmental impacts when the cumulative mileage exceeds 668,240 km.
4. The study introduced the substitution factor method to quantify the reduction in greenhouse gas emissions resulting from the replacement of steel battery box with lightweight alternatives. The results demonstrated that the use of aluminum alloy battery box reduced carbon emissions by 44.4%, with a substitution factor of 0.556, while CF-SMC battery boxes reduced carbon emissions by 34.6%, with a substitution factor of 0.654.

This study provides environmental decision-making basis for the material selection of battery boxes and contributes to the development of lifecycle databases for the power battery industry in new energy vehicles.

### Supplementary Information

Supplementary Information.

## Data Availability

All data generated or analysed during this study are included in this published article (and its Supplementary Information files).
