# Supplementary figures and images for: Environmental impact assessment of battery boxes based on lightweight material substitution
Source: Sci Rep. 2024 Jan 31;14:2594. doi: 10.1038/s41598-024-53238-2 (PMC10830552; doi:10.1038/s41598-024-53238-2)

Supplementary Information

Inventory data of Carbon Fiber Recycling Co. pyrolysis[32,33]


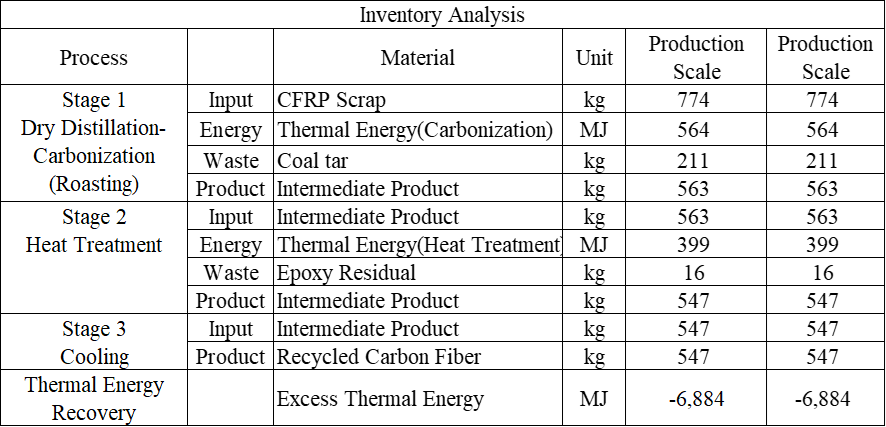

Supplement: Supplementary file 1 — Supplementary Information. [file 41598_2024_53238_MOESM1_ESM.docx]
